# Cesarean section without medical indication and risk of childhood asthma, and attenuation by breastfeeding

**DOI:** 10.1371/journal.pone.0184920

**Published:** 2017-09-18

**Authors:** Shuyuan Chu, Qian Chen, Yan Chen, Yixiao Bao, Min Wu, Jun Zhang

**Affiliations:** 1 The Laboratory of Respiratory Disease, Affiliated Hospital of Guilin Medical University, Guilin, China; 2 MOE-Shanghai Key Laboratory of Children’s Environmental Health, Xinhua Hospital, School of Medicine, Shanghai Jiao Tong University, Shanghai, China; 3 Department of Pediatrics, Xinhua Hospital, School of Medicine, Shanghai Jiao Tong University, Shanghai, China; 4 Department of Traditional Chinese Medicine, Xinhua Hospital, School of Medicine, Shanghai Jiao Tong University, Shanghai, China; 5 School of Public Health, Guilin Medical University, Guilin, China; Liverpool School of Tropical Medicine, UNITED KINGDOM

## Abstract

**Background:**

Previous studies suggest that caesarean section (CS) may increase the risk of asthma in children, but none of them could preclude potential confounding effects of underlying medical indications for CS. We aim to assess the association between CS itself (without medical indications) and risk of childhood asthma.

**Methods:**

We conducted a hospital-based case-control study on childhood asthma with 573 cases and 812 controls in Shanghai. Unconditional logistic regression models in SAS were employed to control for potential confounders.

**Results:**

Our study found that CS without medical indication was significantly associated with elevated asthma risk (adjusted OR = 1.58 [95% CI 1.17–2.13]). However, this risk was attenuated in children fed by exclusive breastfeeding in the first six months after birth (adjusted OR = 1.39 [95% CI 0.92–2.10]). In contrast, the risk was more prominent in children with non-exclusive breastfeeding or bottle feeding (adjusted OR = 1.91 [95% CI 1.22–2.99]).

**Conclusions:**

CS without medical indication was associated with an increased risk of childhood asthma. Exclusive breastfeeding in infancy may attenuate this risk.

## Introduction

Incidence of asthma has increased dramatically worldwide and become a great health burden in children in recent decades [1]. Previous studies suggested that cesarean section (CS) was a risk factor for childhood asthma [2,3]. However, almost all CS were performed for fetal and maternal indications in previous reports [2,3]. And these indications themselves may be risk factors for childhood asthma. For example, fetal growth restriction and pre-term birth are associated with higher likelihood of both CS and childhood asthma [4,5]. The observed association, therefore, might be due to residual confounding by indication even though such fetal and maternal complications were often adjusted in the analyses.

China has a very high CS rate [6]. More than half of the CS are due to maternal or family request without any medical indication (56% of all CS) [7]. Thus, China provides an ideal setting to study if CS itself is associated with the risk of childhood asthma. We conducted this hospital-based case-control study to investigate this issue.

## Methods

From June 2015 to January 2016, children with asthma diagnosed by pediatrician according to the definition of the Global Initiative for Asthma guidelines [8] at the age between 4 to 12 years were recruited in the case group from the Xinhua Hospital, Shanghai, China. The controls were non-asthma patients at the same age range from pediatric outpatient clinic and pediatric surgery clinic. The study protocol was approved by the Institutional Review Board at the Xinhua Hospital. A parental informed consent was obtained for each child. A face-to-face interview was conducted with the parents of both asthmatic children and controls, which included information on parental demographic characteristics, mode of delivery of the child, feeding and environmental exposure. The questionnaire for the control group included a wheezing module for 6–7 years old from the International Study of Asthma and Allergies in Childhood, in order to exclude potential asthmatic children [9].

Information on mode of delivery (CS vs. vaginal delivery) and breastfeeding at the first six month after birth was reported by parents. We further inquired whether the CS was performed due to woman or family request without medical indication, fetal complications, maternal diseases or pregnant complications, or other reasons. The fetal indications included dystocia, fetal distress, suspected macrosomia or fetal growth restriction, fetal malposition, multiple gestation. The maternal indications consisted of severe maternal chronic diseases or pregnancy complications such as congenital heart disease and severe hypertensive disorders in pregnancy. The other reasons for CS included the history of previous CS, placenta praevia, placental abruption, nuchal cord, premature aging of the placenta, and uterine malformation.

We first examined the maternal and infant demographic characteristics in cases and controls. We then explored the associations between CS and the risk of childhood asthma. We further examined the modifiable effect of postpartum breastfeeding on the association between CS and childhood asthma in a stratified analysis. If a covariant changed the association between exposure and outcome by 10% or more, this variable was considered as a potential confounder and was included in the multivariate model. We identified the following potential confounders: maternal education levels (≤9, 10–12, 13–16, or ≥17 years), paternal education levels (≤9, 10–12, 13–16, or ≥17 years), gender (boy/girl), newborn resuscitation (no/yes), and family history of allergic diseases in any of his family members (no/yes). Missing data of the confounders were included as a separate category in the analysis. Unconditional logistic regression models with LOGISTIC procedure in SAS 9.2 (SAS Institute Inc., Cary, North Carolina) were used. The results were presented as odds ratios (OR) and 95% confidence intervals (CI).

## Results

**Fig 1:**
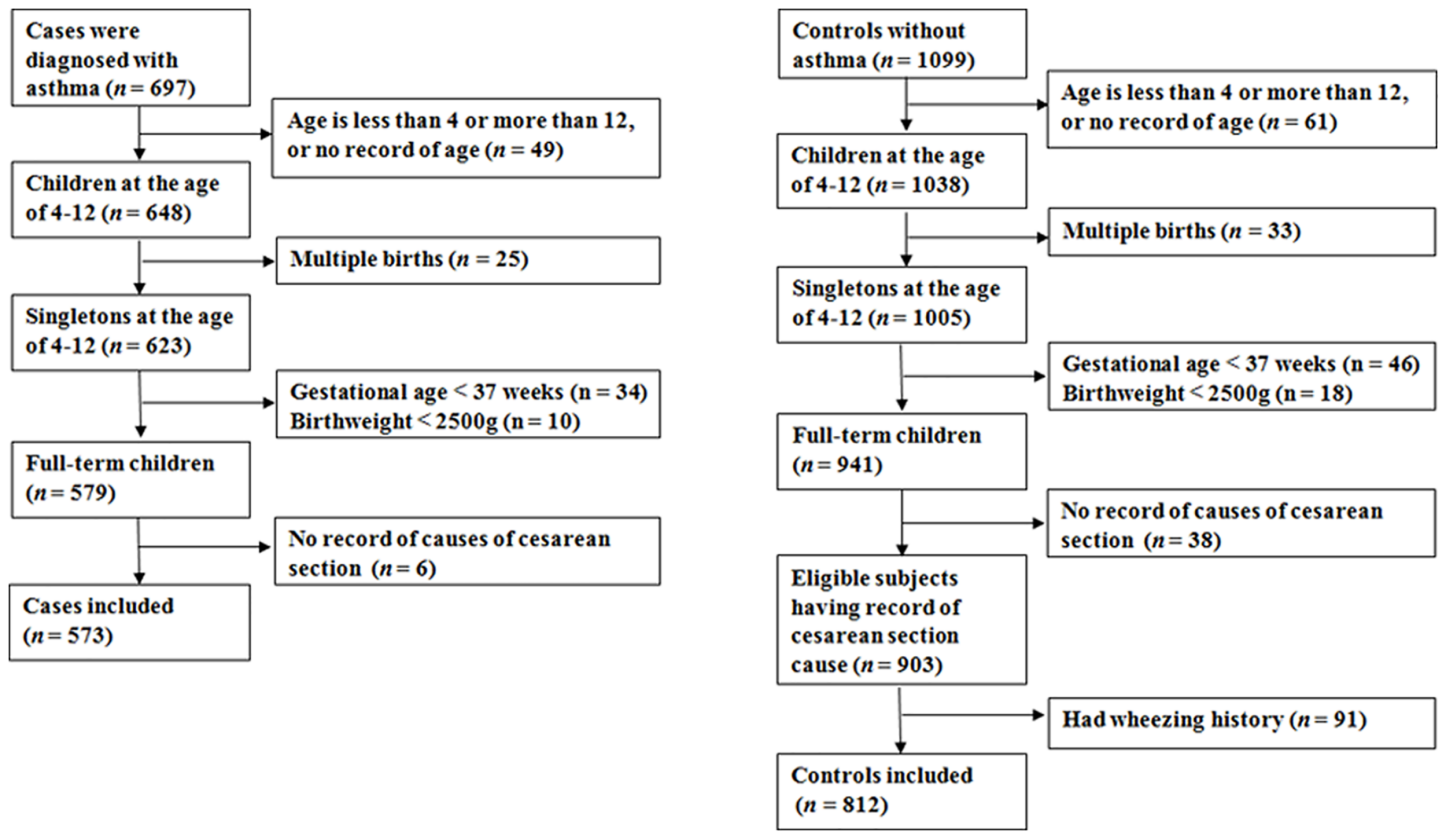
Population flow chart of the case-control study.

Fig 1 illustrates the subject selection process. We excluded cases and controls if they were not at the age between 4 to 12 years or had missing information on age, twins, preterm births or low birth weight, or had no information on CS indications. Controls with history of wheezing were also excluded, leaving a total of 573 cases and 812 controls for final analyses.

The case group had a higher proportion of CS, especially CS without indications, compared with the control groups. The cases were more likely to be boys, younger, and at higher levels of their parents’ education (Table 1).

**Table 1:** Demographic, perinatal and child characteristics in the case-control study.

Characteristics	Case (n = 573)		Control (n = 812)		P value ^a^
	No.	%	No.	%	
**Sex (boys)**	347	61	414	51	<0.01
**Age (yrs)**	226	39	398	49	<0.01
4	207	36	203	25	
5–6	208	36	268	33	
7–8	94	16	176	22	
9–10	50	9	131	16	
11–12	14	2	34	4	
**Family history of allergic diseases**	153	27	161	20	<0.01
**Mode of delivery**					0.03
Vaginal delivery	215	38	363	45	
CS without indications	138	24	145	18	
CS for fetal complications	166	29	228	28	
CS for maternal disorders	16	3	19	2	
CS for other reasons	38	7	57	7	
**Birth weight (g)**					0.87
2500–2999	80	14	115	14	
3000–3499	279	49	399	49	
3500–3999	163	28	236	29	
≥4000	51	9	62	8	
**Newborn resuscitation**	12	2	18	2	0.88
**Feeding in the first 6 months**					0.35
breast feeding	305	53	453	56	
Mixed or exclusive formula feeding	268	47	359	44	
**Passive smoking**	194	34	376	46	<0.01
**Maternal educational level (yrs)**					<0.01
≤9	37	6	149	18	
10–12	71	12	144	18	
13–16	360	63	402	50	
≥17	48	8	40	5	
**Paternal education level (yrs)**					<0.01
≤9	35	6	132	16	
10–12	64	11	150	18	
13–16	347	61	395	49	
≥17	68	12	59	7	

^a^: P values were determined by χ^2^ test.

CS: caesarean section

Table 2 further illustrates that CS without medical indication was associated with an increased risk of childhood asthma after adjusting for potential confounders (adjusted OR = 1.58 [95% CI 1.17–2.13]). When subjects were stratified by feeding pattern within six months after birth, the risk of childhood asthma was higher in children fed by non-exclusive breastfeeding than those fed by exclusive breastfeeding (Table 2).

**Table 2:** Adjusted and unadjusted relative risks of asthma in children exposed to different modes of delivery and stratified by feeding within six months after birth.

Exposure Categories	Case	Control	Unadjusted OR	95% CI	Adjusted OR	95% CI
**All subjects** ^a^						
Vaginal delivery	215	363	Ref.	\	Ref.	\
CS without indications	138	145	1.61	1.21–2.14	1.58	1.17–2.13
CS for fetal complications	166	228	1.23	0.95–1.60	1.15	0.87–1.51
CS for maternal disorders	16	19	1.42	0.72–2.82	1.21	0.58–2.51
CS for other reasons	38	57	1.13	0.72–1.75	1.17	0.74–1.86
**Subjects fed by exclusive breastfeeding** ^b^						
Vaginal delivery	131	220	Ref.	\	Ref.	\
CS without indications	66	79	1.40	0.95–2.08	1.39	0.92–2.10
CS for fetal complications	85	124	1.15	0.81–1.64	1.06	0.73–1.53
CS for maternal disorders	8	8	1.68	0.62–4.58	1.61	0.54–4.81
CS for other reasons	15	22	1.15	0.57–2.29	1.19	0.58–2.45
**Subjects fed by non-exclusive breastfeeding** ^c^						
Vaginal delivery	84	143	Ref.	\	Ref.	\
CS without indications	72	66	1.86	1.21–2.85	1.91	1.22–2.99
CS for fetal complications	81	104	1.33	0.89–1.97	1.23	0.81–1.86
CS for maternal disorders	8	11	1.24	0.48–3.20	1.23	0.45–3.31
CS for other reasons	23	35	1.12	0.62–2.02	1.11	0.60–2.06

^a^: adjusted for paternal education level, and family history of allergic diseases.

^b^: adjusted for maternal education level, paternal education level, and family history of allergic diseases.

^c^: adjusted for paternal education level, and family history of allergic diseases.

CS: caesarean section

The potential interactions between CS and breastfeeding was examined. But no meaningful interactions were found (S1 and S2 Tables). A sensitivity analysis was also conducted by excluding 119 controls from pediatric surgery clinic. The results were similar (adjusted OR = 1.54 [95% CI 1.13–2.09] for all subjects; adjusted OR = 1.36 [95% CI 0.89–2.09] for subjects fed by exclusive breastfeeding; and adjusted OR = 1.81 [95% CI 1.13–2.88] for subjects fed by non-exclusive breastfeeding).

## Discussion

Our study shows that CS without medical indication was associated with an increased risk of childhood asthma. Breastfeeding may attenuate this risk.

Our findings were consistent with that of a meta-analysis which showed that CS modestly increased the risk of childhood asthma (adjusted ORs ranging from 1.11 to 1.31)[3]. Similarly, a population-based data-linkage study with 321,287 term singleton first-born offspring found that in comparison with children born vaginally, offspring born by planned CS were at an increased risk of asthma requiring hospital admission (adjusted hazard ratio = 1.22 [95% CI, 1.11–1.34]) and salbutamol inhaler prescription at age 5 years (adjusted hazard ratio (HR) = 1.13 [95% CI, 1.01–1.26]) [10]. Interestingly, Almqvist et al. found an increased risk of asthma among children delivered by emergency CS but not elected CS [11], whereas Black et al. showed that the risk of asthma was somewhat greater among children delivered by planned CS (adjusted HR = 1.24 [95% CI, 1.09–1.42]) than those by unscheduled CS (adjusted HR = 1.18 [95% CI 1.05–1.33]) [12]. The inconsistent findings may be due to different subjects and potential confounding effect of underlying medical indications for CS. In our study, the risk of childhood asthma is a little higher than those previously reported, which may be due to the exclusion of potential confounding by indications in our study. In addition, we excluded children who had wheezing history from controls, since those children may be potential asthma patients.

The CS without medical indication may increase the risk of childhood asthma due to exposure to different microbiota during delivery in comparison to vaginal births. Fetuses delivered by CS are mainly exposed to microbiota that is predominately on maternal skin after birth, instead of those in maternal vagina [13].This altered microbial types and colonization in early life of the child may alter natural development of immune system and then promote the development of immune-mediated asthma [13].This might be supported by the evidence that manually exposing newborns delivered by CS to maternal vaginal microbes may partially restore normal microbiota of these infants [14].Our study provides further support to this microbiota-related mechanism by showing the protective effect of exclusive breastfeeding in infancy on the risk of childhood asthma since breastfeeding could prevent allergy through regulating infant gut barrier function and microbiota [15]. Moreover, breastfeeding may also transmit immune-modulatory cytokines, immunoglobulins and chemokines to infants, which could promote development of child immune system and stimulation of alveolarization [16,17].

We acknowledge that our study has limitations. Perhaps the most significant limitation of the study is that it drew on a hospital-based sample. Wealthier, better-educated families might be more likely to bring their children with asthma symptoms to hospital. These families may also be more likely to request CS at birth. Thus, even though we have adjusted for maternal and paternal education, it is still possible that residual confounding may have affected our results. The true underlying association may be weaker than that in our study. Second, information on CS and its indications were self-reported. A previous study demonstrated that the accuracy of maternal recall of CS 3 to 9 years ago was 100%, and maternal recall of severe obstetric complications was also rather reliable [18]. Moreover, the average prevalence of CS (58%) and the rate of CS without medical indications (20%) in our study was almost the same as that in a previous report, where CS prevalence and the rate of CS on maternal request were 56% and 20%, respectively [7]. Therefore, the self-reported CS and its indications may be reasonably accurate in our study. Third, the data on feeding in infancy was recalled by parents. In our study, the prevalence of exclusive breastfeeding was similar to that previously reported in Shanghai, around 50% [19]. Thus, this recall may not be seriously biased.

In conclusion, CS without medical indication is associated with an increased risk of childhood asthma. Our study avoided the challenge of potential residual confounding by CS indications and illustrated a clear relationship. CS may have contributed to the increased prevalence of childhood asthma. Fortunately, breastfeeding may attenuate this risk. This finding may have important clinical and public health implications.

## Supporting information

S1 Table Interaction between caesarean section and breastfeeding. (DOCX)

S2 Table Interaction between caesarean section and breastfeeding. (DOCX)

S1 Checklist Checklist of items that included in this study. (DOCX)

## References

[1] Wong GW, Leung TF, Ko FW. Changing prevalence of allergic diseases in the Asia-pacific region. Allergy Asthma Immunol Res 2013;5:251–257. doi: 10.4168/aair.2013.5.5.251 24003381 10.4168/aair.2013.5.5.251 PMC3756171

[2] Bager P, Wohlfahrt J, Westergaard T. Caesarean delivery and risk of atopy and allergic disease: meta-analyses. Clin Exp Allergy 2008;38:634–642. doi: 10.1111/j.1365-2222.2008.02939.x 18266879 10.1111/j.1365-2222.2008.02939.x

[3] Thavagnanam S, Fleming J, Bromley A, Shields MD, Cardwell CR. A meta-analysis of the association between Caesarean section and childhood asthma. Clin Exp Allergy 2008;38:629–633. doi: 10.1111/j.1365-2222.2007.02780.x 18352976 10.1111/j.1365-2222.2007.02780.x

[4] Turner SW, Campbell D, Smith N, Craig LC, McNeill G, Forbes SH, et al Associations between fetal size, maternal {alpha}-tocopherol and childhood asthma. Thorax 2010;65:391–397. doi: 10.1136/thx.2008.111385 20435859 10.1136/thx.2008.111385

[5] Tedner SG, Örtqvist AK, Almqvist C. Fetal growth and risk of childhood asthma and allergic disease. Clin Exp Allergy 2012;42:1430–1447. doi: 10.1111/j.1365-2222.2012.03997.x 22994341 10.1111/j.1365-2222.2012.03997.x PMC3564398

[6] Feng XL, Xu L, Guo Y, Ronsmans C. Factors influencing rising caesarean section rates in China between 1988 and 2008. Bull World Health Organ 2012;90:30–9,39A doi: 10.2471/BLT.11.090399 22271962 10.2471/BLT.11.090399 PMC3260572

[7] Zhang J, Liu Y, Meikle S, Zheng J, Sun W, Li Z. Cesarean delivery on maternal request in southeast China. Obstet Gynecol 2008;111: 1077–1082. doi: 10.1097/AOG.0b013e31816e349e 18448738 10.1097/AOG.0b013e31816e349e

[8] http://www.ginasthma.org/.

[9] Asher MI, Keil U, Anderson HR, Beasley R, Crane J, Martinez F, et al International Study of Asthma and Allergies in Childhood (ISAAC): rationale and methods. Eur Respir J 1995;8:483–491. 7789502 10.1183/09031936.95.08030483

[10] Leung JY, Li AM, Leung GM, Schooling CM. Mode of delivery and childhood hospitalizations for asthma and other wheezing disorders. Clin Exp Allergy 2015;45:1109–1117. doi: 10.1111/cea.12548 25845852 10.1111/cea.12548

[11] Almqvist C, Cnattingius S, Lichtenstein P, Lundholm C. The impact of birth mode of delivery on childhood asthma and allergic diseases—a sibling study. Clin Exp Allergy. 2012;42(9):1369–76. doi: 10.1111/j.1365-2222.2012.04021.x 22925323 10.1111/j.1365-2222.2012.04021.x PMC3564396

[12] Black M, Bhattacharya S, Philip S, Norman JE, McLernon DJ. Planned Repeat Cesarean Section at Term and Adverse Childhood Health Outcomes: A Record-Linkage Study. PLoS Med. 2016;13(3):e1001973 doi: 10.1371/journal.pmed.1001973 26978456 10.1371/journal.pmed.1001973 PMC4792387

[13] Kaplan JL, Shi HN, Walker WA. The role of microbes in developmental immunologic programming. Pediatr Res 2011;69:465–472. doi: 10.1203/PDR.0b013e318217638a 21364495 10.1203/PDR.0b013e318217638a

[14] Dominguez-Bello MG, De Jesus-Laboy KM, Shen N, Cox LM, Amir A, Gonzalez A, et al Partial restoration of the microbiota of cesarean-born infants via vaginal microbial transfer. Nat Med 2016;22:250–253. doi: 10.1038/nm.4039 26828196 10.1038/nm.4039 PMC5062956

[15] Munblit D, Verhasselt V. Allergy prevention by breastfeeding: possible mechanisms and evidence from human cohorts. Curr Opin Allergy Clin Immunol 2016;16(5):427–33. doi: 10.1097/ACI.0000000000000303 27518839 10.1097/ACI.0000000000000303

[16] Soto-Ramirez N, Karmaus W, Yousefi M, Zhang H, Liu J, Gangur V. Maternal immune markers in serum during gestation and in breast milk and the risk of asthma-like symptoms at ages 6 and 12 months: a longitudinal study. Allergy Asthma Clin Immunol.2012;8(1):11 doi: 10.1186/1710-1492-8-11 22805009 10.1186/1710-1492-8-11 PMC3536636

[17] Politis I, Chronopoulou R. Milk peptides and immune response in the neonate. Adv Exp Med Biol. 2008;606:253–269. doi: 10.1007/978-0-387-74087-4_10 18183933 10.1007/978-0-387-74087-4_10

[18] Sou SC, Chen WJ, Hsieh WS, Jeng SF. Severe obstetric complications and birth characteristics in preterm or term delivery were accurately recalled by mothers. J Clin Epidemiol 2006;59:429–435. doi: 10.1016/j.jclinepi.2005.08.010 16549266 10.1016/j.jclinepi.2005.08.010

[19] Ma JQ, Zhou LL, Hu YQ, Liu JR, Liu SS, Zhang J, et al A summary index of infant and child feeding practices is associated with child growth in urban Shanghai. BMC Public Health. 2012;12:568 doi: 10.1186/1471-2458-12-568 22839527 10.1186/1471-2458-12-568 PMC3487749

