# Supplementary material for: Cesarean section without medical indication and risk of childhood asthma, and attenuation by breastfeeding
Source: PLoS One. 2017 Sep 18;12(9):e0184920. doi: 10.1371/journal.pone.0184920 (PMC5602659; doi:10.1371/journal.pone.0184920)
Supplement: S1 Table — (DOCX) [file pone.0184920.s001.docx]

S1 Table. Interaction between caesarean section and breastfeeding.

|  | **Unadjusted estimated** | | **Adjusted estimated ^a^** | |
| --- | --- | --- | --- | --- |
| **Exposure** | **beta** | **P value** | **beta** | **P value** |
| CS | 0.23 | 0.13 | 0.18 | 0.25 |
| Breastfeeding | -0.13 | 0.35 | -0.07 | 0.62 |
| CS* Breastfeeding | 0.15 | 0.52 | 0.17 | 0.47 |

a: adjusted for maternal education level, paternal education level, and family history of allergic diseases.

CS: caesarean section
