# Supplementary material for: Cesarean section without medical indication and risk of childhood asthma, and attenuation by breastfeeding
Source: PLoS One. 2017 Sep 18;12(9):e0184920. doi: 10.1371/journal.pone.0184920 (PMC5602659; doi:10.1371/journal.pone.0184920)
Supplement: S2 Table — (DOCX) [file pone.0184920.s002.docx]

S2 Table. Interaction between caesarean section and breastfeeding.

| **Model** | | **Unadjusted estimated** | | **Adjusted estimated ^a^** | |
| --- | --- | --- | --- | --- | --- |
|  |  | **OR** | **95% CI** | **OR** | **95% CI** |
| OR_cs_  OR_breastfeeding_ | | 1.25 | 0.94-1.68 | 1.20 | 0.88-1.62 |
|  |  | 0.88 | 0.66-1.16 | 0.93 | 0.70-1.24 |
| OR_cs_=1 and OR_breastfeeding_=1 | OR_cs_=1, OR_breastfeeding_=0 | 1.45 | 0.69-3.03 | 1.42 | 0.66-3.07 |
|  | OR_cs_=0, OR_breastfeeding_=1 | 1.01 | 0.49-2.08 | 1.11 | 0.52-2.35 |

a: adjusted for maternal education level, paternal education level, and family history of allergic diseases.

CS: caesarean section
